# A retrospective analysis of outpatient use of small-molecule targeted inhibitors for lymphoma across six regions of China (2016–2022)

**DOI:** 10.3389/fphar.2026.1784805

**Published:** 2026-03-04

**Authors:** Bo Chen, Li-Ying Chen, Ping Chen, Chen Wang, Chang-Wei Yang, Yan Hu, Ran Wei, Liu-Cheng Li, Zhen-Ling Fu

**Affiliations:** 1 Department of Pharmacy, Sir Run Run Shaw Hospital, School of Medicine, Zhejiang University, Hangzhou, Zhejiang, China; 2 Department of Pharmacy, The Quzhou Affiliated Hospital of Wenzhou Medical University, Quzhou People’s Hospital, Quzhou, Zhejiange, China

**Keywords:** lymphoma, outpatient prescription trends, pharmacoeconomics, small-molecule targeted inhibitors, targeted therapy

## Abstract

**Objective:**

This study aimed to assess national trends in prescription volumes, drug expenditures, and the pharmacoeconomic rationality of small-molecule targeted inhibitors used for lymphoma treatment among outpatients in China between 2016 and 2022.

**Methods:**

Outpatient prescription data for patients diagnosed with lymphoma were obtained from the Hospital Prescription Analysis Cooperative Project database, which includes 77 hospitals distributed across six major regions of China. Annual trends in prescription volume and corresponding drug expenditures were examined. Pharmacoeconomic indicators associated with small-molecule targeted inhibitors were further analyzed to evaluate their cost-effectiveness and utilization patterns. Patient demographic characteristics, regional distribution, and categories of small-molecule targeted inhibitors were also analyzed.

**Results:**

Prescription volumes and amounts for small-molecule targeted inhibitors in lymphoma treatment have increased annually. Furthermore, their use is supported by pharmacoeconomic evidence indicating rational and efficient medication utilization. There was a statistically significant increase in total prescriptions (*P*_*1*_ < 0.005) and overall medication expenditures (*P*_*2*_ < 0.005).

**Conclusion:**

Between 2016 and 2022, the prescription volume of small-molecule targeted inhibitors for lymphoma increased annually, indicating their expanding clinical use. Since 2020, despite continued growth in prescriptions, drug costs have risen at a slower rate than prescriptions. This reflects that medical insurance negotiation and centralized procurement policies have effectively reduced economic burden without limiting their access to these inhibitors. Pharmacoeconomic indicators also confirm that the use of these drugs has been both reasonable and efficient, allowing for increased drug utilization while reducing financial strain.

## 1 Introduction

Lymphoma is a relatively rare malignancy and is mainly classified into Hodgkin’s lymphoma and non-Hodgkin’s lymphoma. Because lymphoma often involves systemic dissemination of tumor cells and is generally not amenable to surgical treatment, clinical management relies primarily on radiotherapy and chemotherapy (Elliott et al., 2019; Ng et al., 2025; Zhang C et al., 2023; Castellino et al., 2022). With disease progression and continuous advances in molecular biology and related fields, targeted therapy has gradually become an important component of lymphoma treatment. Recent progress in cell biology has further promoted the development and clinical application of targeted therapies for lymphoma.

The identification of novel drug targets has opened new avenues for therapeutic intervention, including Bruton’s Tyrosine Kinase (BTK) inhibitors (Cool et al., 2024) and Vascular Endothelial Growth Factor (VEGF)/Vascular Endothelial Growth Factor Receptor (VEGFR) inhibitors (Mabeta et al., 2022). At present, the development of anti-lymphoma targeted drugs with high selectivity, low toxicity, and favorable efficacy remains a major focus of research. Among these therapeutic strategies, BTK-related alterations represent the most frequently targeted mechanism in lymphoma treatment. In addition, mutations in the Anaplastic Lymphoma Kinase (ALK) gene have been identified in a subset of lymphoma patients (Medical Oncology Branch of Chinese International Exchange and Promotion Association for Medical and Healthcare, et al., 2023).

Moreover, because lymphoma is a rare disease with a relatively low incidence rate, existing clinical research is largely limited to case-based analyses, and systematic studies based on large-scale datasets are still lacking. This study conducts a systematic analysis of outpatient medication use among lymphoma patients using prescription data from 77 hospitals located in six major cities across China. Real-world research on the use of targeted therapies for lymphoma can provide valuable evidence for clinical practice, while comprehensive evaluation of medication utilization may further promote rational drug use in clinical settings.

In addition, reform of the medical insurance system has become an important topic in current health policy research. Given the limited total resources of medical insurance, it is necessary to achieve optimal health outcomes for the majority of the population at a lower overall cost, thereby improving the efficiency of the medical insurance system. To this end, medical insurance policies should prioritize treatment options that achieve equivalent therapeutic effects at lower costs. At present, systematic evaluations of the economic characteristics of small-molecule targeted inhibitors used for lymphoma are lacking. This study assesses the use of small-molecule targeted inhibitors for lymphoma from a pharmacoeconomic perspective, providing evidence to support decision-making by medical insurance agencies and health authorities.

### 1.1 Methods

#### 1.1.1 Study design

This work was conducted as a cross-sectional analysis based on prescription data collected from hospital outpatient records.

#### 1.1.2 Data source and study sample

Prescription data were extracted from the Hospital Prescription Analysis Cooperative Project database (Chen, et al., 2025a; Chen et al., 2025b; Li et al., 2021; Wang et al., 2025; Dimopoulos et al., 2023), which has been widely used in our previous studies as well as in numerous epidemiological investigations across China. The database contains outpatient prescription records from participating hospitals and covers 40 randomly selected days each year. Prescriptions were included in the analysis if they met the following criteria: (i) contained keywords such as “tinib”; (ii) were issued to patients diagnosed with lymphoma, regardless of diagnostic criteria, disease subtype, or severity; (iii) included at least one medication related to lymphoma treatment, whether as an initial or renewal prescription, and were issued between 1 January 2016, and 30 June 2022; and (iv) originated from hospitals located in Beijing, Chengdu, Guangzhou, Hangzhou, Shanghai, or Zhengzhou, all of which continuously participated in the project throughout the study period. For temporal reference, the first half of each year was denoted as “s” and the second half as “x” (for example, “2016s” represents January to June 2016, and “2016x” represents July to December 2016). From each eligible prescription, the following information was extracted: prescription code, patient sex and age, year of issuance, hospital location, hospital code, diagnosis, generic drug names, and the cost of each medication. As this was a retrospective study, no identifiable personal information was involved; all data were anonymized and analyzed using coded identifiers only. According to the regulations of the ethics committee of our institution, ethical approval was not required for this study. The analysis was conducted in accordance with the ethical principles of the World Medical Association and the Declaration of Helsinki. A total of 1,232 outpatient cases were initially identified, and final inclusion was determined after applying the predefined inclusion and exclusion criteria.

#### 1.1.3 Assessment of medicine use

Medicine use was assessed based on the number of prescriptions, regardless of whether they were newly issued or refilled, with each prescription corresponding to one outpatient visit. The total cost was calculated in Chinese Yuan (CNY) by summing the prices of all included medicines. Trends in annual prescription volumes and associated costs were analyzed and further stratified by sex, age group, medicine class, and individual drug. Prescriptions were categorized according to medications used for lymphoma treatment and those prescribed for accompanying conditions. The drugs included Vascular Endothelial Growth Factor Receptor–Tyrosine Kinase Inhibitors (VEGFR-TKIs), such as apatinib; Anaplastic Lymphoma Kinase–Tyrosine Kinase Inhibitors (ALK-TKIs), including alectinib and ceritinib; and Bruton’s Tyrosine Kinase–Tyrosine Kinase Inhibitors (BTK-TKIs), such as orelabrutinib, ibrutinib, and zanubrutinib.

#### 1.1.4 Data analysis

Statistical significance was defined as P < 0.05. All prescription data were processed using Microsoft Office Excel 2016 (Microsoft Corp., Redmond, WA, USA). Figures were generated using GraphPad Prism version 9. Statistical analyses were performed using R software version 4.3.2 (http://www.R-project.org).

## 2 Results

### 2.1 Demographic characteristics of outpatients using small-molecule targeted inhibitors and trends in overall usage

**FIGURE 1:**
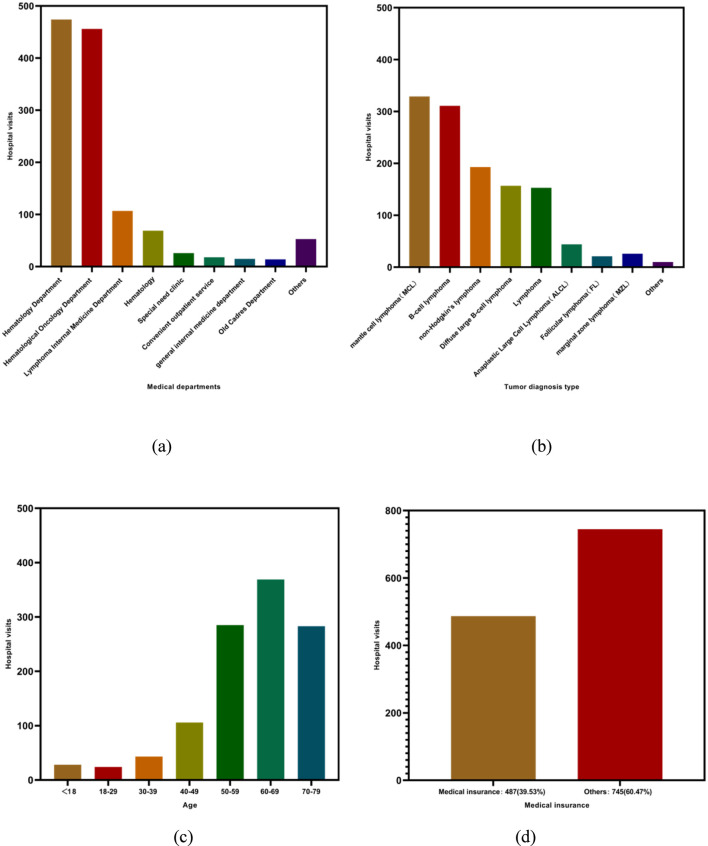
Distribution of lymphoma outpatients by clinical department, diagnosis type, age group, and medical insurance status: **(a)** medical department; **(b)** tumor diagnosis type; **(c)** age; **(d)** medical insurance.

Demographic characteristics of lymphoma outpatients by sex, age group, city, and hospital level are showed in Table 1. Regarding the clinical departments from which patients originated, the majority were associated with oncology-related services. The hematology department accounted for the largest proportion, with 474 patients (38.5%), followed by the oncology department with 456 patients (37.0%). Patients from the lymphoma oncology department and hematological oncology department accounted for 107 (8.7%) and 69 (5.6%) cases, respectively. Other sources included the special medical care department with 26 patients (2.10%), the convenient outpatient department with 18 patients (1.50%), general internal medicine with 15 patients (1.20%), the elderly cadre department with 14 patients (1.10%), and other departments with 53 patients (4.3%) in Figure 1a.

**TABLE 1:** Demographic characteristics of lymphoma outpatients by sex, age group, city, and hospital level.

​	2016s	2016x	2017s	2017x	2018s	2018x	2019s	2019x	2020s	2020x	2021s	2021x	2022s	*P*_1_	*P*_2_
City															
Beijing	0	1	0	0	1	1	6	6	14	16	25	17	22	0.01	0.162
Chengdu	0	0	0	0	0	0	0	0	0	0	0	0	3	—	—
Guangzhou	1	1	2	0	4	6	44	50	55	48	87	131	202	0.003	0.689
Hangzhou	0	0	0	0	0	0	5	21	29	29	30	44	75	0.008	0.023
Shanghai	0	0	0	0	2	0	4	9	13	24	25	42	21	0.01	0.201
Zhengzhou	0	0	0	0	0	0	7	15	15	14	16	28	21	0.008	0.125
Hospital level															
Second-class hospital	0	0	0	0	0	0	0	0	0	0	0	0	0	—	—
Tertiary hospitals	1	2	2	0	7	7	66	101	126	131	183	262	344	0.003	—
Sex															
Male	1	2	2	0	6	5	42	64	80	98	127	186	229	0.003	0.957
Female	0	0	0	0	1	2	24	37	46	33	56	76	115	0.004	0.957

*P*_*1*_ refers to the p-value for the trend in the number of prescriptions, assessed using the Mann-Kendall trend test. *P*_*2*_ refers to the p-value for the trend in prescriptions, assessed using the Cochran-Armitage trend test. When the data volume is fewer than three points, *P*_*1*_ and *P*_*2*_ is not feasible.

In terms of diagnostic classification, some patients were recorded under broad diagnostic categories, including lymphoma (153 cases, 12.42%) and non-Hodgkin’s lymphoma (193 cases, 15.67%). Others were classified according to specific pathological subtypes, including Mantle Cell Lymphoma (MCL) with 329 cases (26.70%), B-cell lymphoma with 311 cases (25.24%), Diffuse Large B-Cell Lymphoma with 157 cases (12.74%), Anaplastic Large Cell Lymphoma (ALCL) with 44 cases (3.57%), Follicular Lymphoma (FL) with 21 cases (1.70%), Marginal Zone Lymphoma (MZL) with 26 cases (2.11%), and other subtypes with 10 cases (0.81%).

Based on diagnostic type analysis, non-Hodgkin’s lymphoma accounted for the overwhelming majority of cases. Except for the general diagnosis of lymphoma (153 cases, 12.42%), which may include a small proportion of Hodgkin’s lymphoma, all remaining diagnostic categories corresponded to non-Hodgkin’s lymphoma or its subtypes. Accordingly, the proportion of non-Hodgkin’s lymphoma cases was at least 87.58% in Figure 1b.

With respect to age distribution, patients aged over 50 years accounted for 76.05% of the total population. Specifically, 285 patients (23.13%) were aged 50–59 years, 369 patients (29.95%) were aged 60–69 years, and 283 patients (22.97%) were aged 70–79 years. Among patients younger than 50 years, 28 (2.27%) were under 18 years of age in Figure 1c, 24 (1.95%) were aged 18–29 years, 43 (3.49%) were aged 30–39 years, and 106 (8.60%) were aged 40–49 years. Regarding medical insurance status, 487 patients (39.53%) were covered by medical insurance, while 745 patients (60.47%) fell into other categories in Figure 1d.

### 2.2 Prescriptions and expenditures of small-molecule targeted inhibitors for lymphoma

**FIGURE 2:**
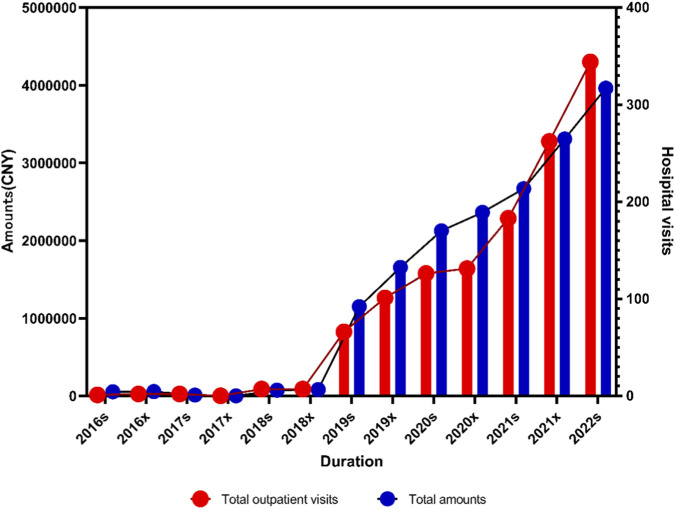
Trends in outpatient visits and total prescription expenditures for small-molecule targeted inhibitors targeting lung cancer from 2016s to 2022s.

Figure 2 shows the total number of prescriptions and overall drug expenditures increased steadily over the study period. Before 2018x, the growth rate was relatively slow. After 2018x, several newly developed targeted drugs for lymphoma were approved for clinical use or included in the medical insurance reimbursement list, leading to a rapid increase in both prescription volume and total expenditure. This trend indicates that the clinical use of small-molecule inhibitors for lymphoma has become increasingly widespread. Although both prescription volume and total expenditure continued to rise, the growth rate of prescription volume was higher than that of total expenditure, suggesting a gradual reduction in the economic burden on patients. The increase in prescription volume was statistically significant (*P*_*1*_ = 0.003), as was the increase in prescription expenditure (*P*_*2*_ < 0.005).

### 2.3 Prescriptions and expenditures of general ALK, BTK, VEGFR inhibitors from 2016s to 2022s. Inhibitors for lymphoma

**FIGURE 3:**
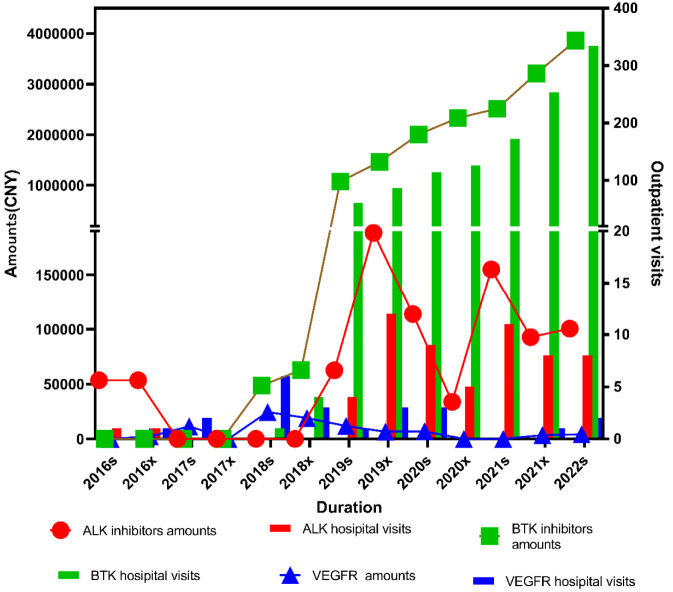
Trends in outpatient visits and prescription expenditures of ALK, BTK, and VEGFR inhibitors for lymphoma from 2016s to 2022s.

Figure 3 shows the prescription volume and total expenditure of three categories of small-molecule inhibitors for lymphoma are presented in the figure. Among these, BTK inhibitors accounted for the largest proportion in both prescription volume and total expenditure, followed by ALK inhibitors, while VEGFR inhibitors ranked third. For ALK inhibitors, changes in total expenditure were statistically significant (*P*_*1*_ = 0.095, *P*_*2*_ = 0.007), whereas changes in prescription volume showed a similar trend (*P*_*1*_ = 0.095, *P*_*2*_ = 0.007). For BTK inhibitors, both total expenditure (*P*_*1*_ = 0.004, *P*_*2*_ < 0.005) and prescription volume (*P*_*1*_ = 0.004, *P*_*2*_ < 0.005) increased significantly over time. In contrast, for VEGFR inhibitors, neither total expenditure (*P*_*1*_ = 0.649, *P*_*2*_ < 0.005) nor prescription volume (*P*_*1*_ = 1.000) showed a statistically significant upward trend.

### 2.4 Prescriptions and expenditures of different BTK inhibitors for lymphoma from 2016s to 2022s

**FIGURE 4:**
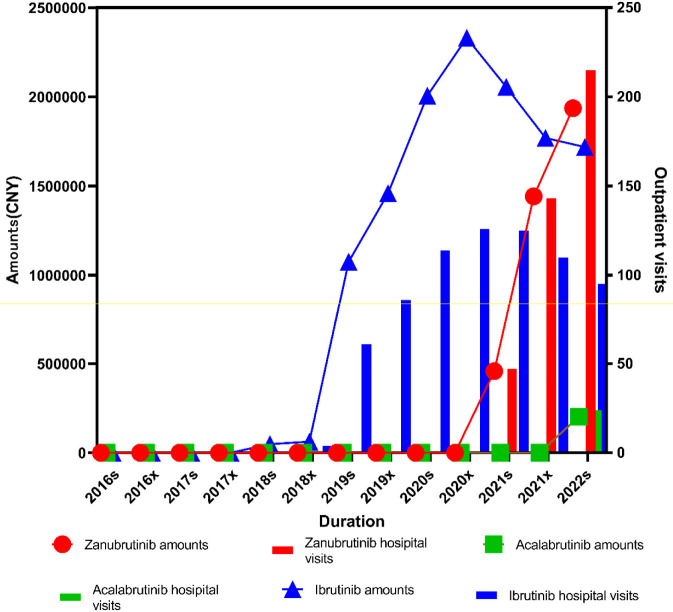
Trends in outpatient visits and prescription expenditures of BTK inhibitors (zanubrutinib, acalabrutinib, and ibrutinib) for lymphoma from 2016s to 2022s.

Overall, the market for BTK inhibitors remained at a relatively low level before 2018. Following the approval of ibrutinib in China in August 2017, both the prescription volume and total expenditure of ibrutinib increased rapidly between 2018 and 2020. After zanubrutinib was approved in China in September 2020, the prescription volume and expenditure of zanubrutinib and orelabrutinib also showed a marked upward trend. In 2021, the prescription volume of zanubrutinib exceeded that of ibrutinib, although its total expenditure remained lower. By 2022, both the prescription volume and expenditure of zanubrutinib surpassed those of ibrutinib, ranking first among BTK inhibitors. Orelabrutinib showed no reported prescription volume or expenditure prior to 2022 and appeared for the first time in that year, with both indicators beginning to increase in Figure 4.

For ibrutinib, statistically significant changes were observed in both prescription volume (*P*_*1*_ = 0.023) and expenditure (*P*_*2*_ < 0.005). Ibrutinib was launched in China in 2017 and included in the medical insurance reimbursement list in 2018. Since then, its prescription volume and expenditure increased rapidly and remained at relatively high levels before peaking in 2021, followed by a gradual decline. In contrast, zanubrutinib demonstrated rapid growth in both prescription volume and expenditure after its launch in 2020, exceeding those of ibrutinib after 2021. Orelabrutinib, approved in China in December 2020, exhibited a relatively slower growth trend.

Ibrutinib is a first-generation BTK inhibitor, whereas zanubrutinib and orelabrutinib belong to the second generation. With the introduction of second-generation BTK inhibitors, the utilization and prescription volume of first-generation agents declined markedly. Among second-generation BTK inhibitors, orelabrutinib showed rapid growth, while the growth rate of zanubrutinib was comparatively slower.

### 2.5 Prescriptions and expenditures of different ALK inhibitors for lymphoma from 2016s to 2022s

**FIGURE 5:**
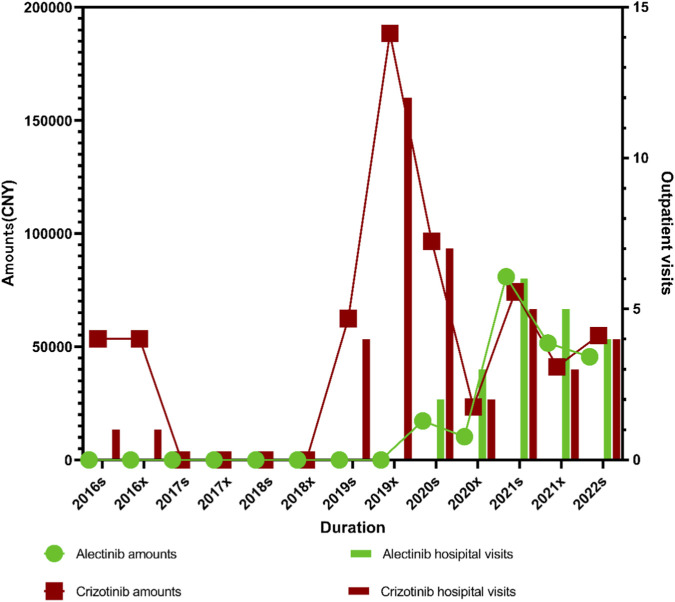
Trends in outpatient visits and prescription expenditures of ALK inhibitors (alectinib and crizotinib) for lymphoma from 2016s to 2022s.

For alectinib, statistically significant increases were observed in both expenditure (*P*_*1*_ = 0.045, *P*_*2*_ < 0.005) and prescription volume (*P*_*1*_ = 0.045, *P*_*2*_ = 0.031). For crizotinib, changes in expenditure (*P*_*1*_ = 0.28761, *P*_*2*_ < 0.005) and prescription volume (*P*_*1*_ = 0.219, *P*_*2*_ = 0.031) were less pronounced. The prescription volume and expenditure of crizotinib reached their peak in 2019 and subsequently declined gradually. After 2018, the launch of alectinib in China and its inclusion in the medical insurance reimbursement list led to a steady increase in its clinical use. In contrast, crizotinib entered the Chinese market earlier, in 2013, and was included in the medical insurance reimbursement list in 2018, resulting in only a modest increase after 2019. With the introduction of the second-generation ALK inhibitor alectinib, both the prescription volume and expenditure of the first-generation ALK inhibitor crizotinib declined rapidly. After 2021, alectinib surpassed crizotinib in both prescription volume and total expenditure, becoming the dominant ALK inhibitor used in lymphoma treatment in Figure 5.

### 2.6 Prescriptions and expenditures of VEGFR inhibitors for lymphoma from 2016s to 2022s

**FIGURE 6:**
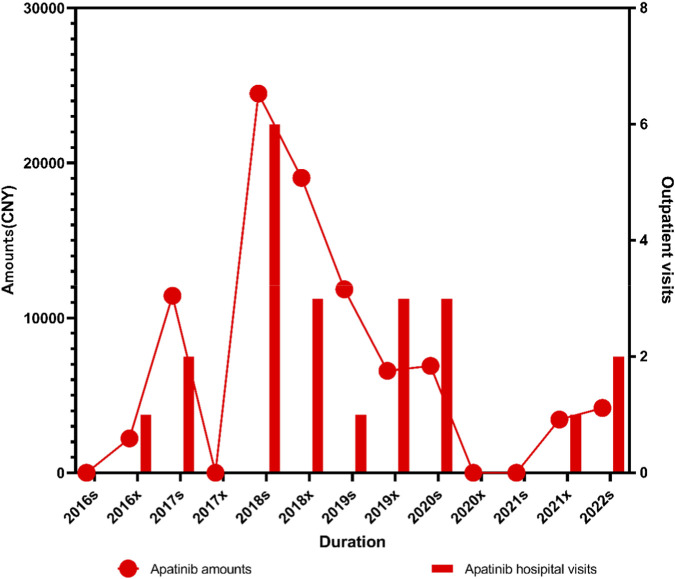
Trends in outpatient visits and prescription expenditures of the VEGFR inhibitor apatinib for lymphoma from 2016s to 2022s.

The time-dependent trend of VEGFR inhibitors showed no clear or consistent pattern over the study period (*P*_*1*_ = 1.000, *P*_*2*_ < 0.005). Overall, no statistically significant time trend was observed, and the fluctuation in prescription volume and expenditure was relatively limited. VEGFR inhibitors reached a peak in 2018. Following the inclusion of apatinib in the medical insurance reimbursement list in 2017, both the prescription volume and total expenditure of apatinib increased rapidly in 2018 and then reached a maximum. Thereafter, the use of VEGFR inhibitors, represented primarily by apatinib, exhibited a fluctuating but generally stable pattern without an obvious long-term trend (*P*_*1*_ = 0.649) in Figure 6.

### 2.7 Pharmacoeconomics and rationality of small-molecule inhibitor use in lymphoma treatment

**FIGURE 7:**
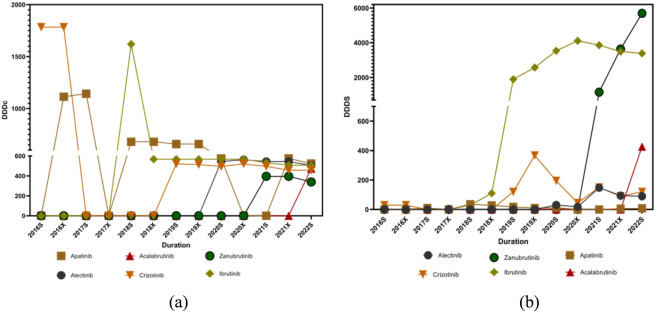
Pharmacoeconomic trends of small-molecule targeted inhibitors for lymphoma from 2016s to 2022s: **(a)** DDDc; **(b)** DDDs.

The Defined Daily Dose (DDD) values for small-molecule targeted inhibitors used in lymphoma treatment were obtained primarily from the World Health Organization (WHO) database. The DDD values applied in this study were as follows: acalabrutinib, 0.2 g; zanubrutinib, 0.32 g; ibrutinib, 0.42 g; crizotinib, 0.5 g; alectinib, 1.2 g; and apatinib, 1.25 g. Analysis of total dosage divided by DDDs revealed a clear upward trend for most small-molecule targeted inhibitors used in lymphoma treatment. The increase in DDDs indicates expanding clinical adoption of these therapies, reflecting both greater physician acceptance and improved patient access in Figure 7.

In contrast, the Defined Daily Drug Cost (DDDc) showed a generally declining trend for most small-molecule targeted inhibitors over the study period in Figure 7. This decrease in DDDc suggests a reduction in patients’ economic burden, likely attributable to medical insurance negotiations and pricing policies in China. Notably, the DDDc of ibrutinib declined significantly after 2020, while the DDDc of zanubrutinib remained relatively low, which may partially explain its rapid increase in market share. For DDDc, no statistically significant time trends were observed for alectinib (*P*_*1*_ = 0.126, *P*_*2*_ < 0.005), apatinib (*P*_*1*_ = 0.288, *P*_*2*_ < 0.005), or crizotinib (*P*_*1*_ = 0.879, *P*_*2*_ < 0.005), whereas a significant decline was observed for ibrutinib (*P*_*1*_ = 1.000, *P*_*2*_ = 0.008). Regarding DDDs, statistically significant increases were identified for alectinib (*P*_*1*_ = 0.045, *P*_*2*_ = 0.512) and ibrutinib (*P*_*1*_ = 0.023, *P*_*2*_ = 0.035), while no significant trends were observed for apatinib (*P*_*1*_ = 0.879, *P*_*2*_ < 0.005) or crizotinib (*P*_*1*_ = 0.219, *P*_*2*_ = 0.339).

## 3 Discussion

This study is the first to systematically examine the utilization patterns of small-molecule targeted inhibitors for lymphoma patients across six major regions of China, revealing rapid developmental trends and notable changes in clinical practice within this field.

Between 2016 and 2022, the use of small-molecule targeted inhibitors for lymphoma demonstrated a pronounced growth trend, particularly after 2018, when an accelerated increase became evident. This shift can be attributed to several factors. First, during the period from 2017 to 2020, innovative agents such as ibrutinib and zanubrutinib were successively approved for use in China, substantially expanding available therapeutic options. Second, the wider adoption of molecular diagnostic technologies enabled more patients to receive precise targeted treatments. Third, the continuous optimization of China’s medical insurance reimbursement policies allowed several high-cost innovative drugs to undergo price negotiations and gain insurance coverage, thereby improving drug accessibility.

Although total drug expenditures increased during the study period, DDDc showed a gradual downward trend. Moreover, the growth rate of prescription volume exceeded that of total costs, suggesting a reduction in the financial burden on patients. This pattern is closely associated with national medical insurance negotiation policies implemented by the National Healthcare Security Administration, which effectively reduced the prices of innovative drugs and enabled broader patient access, reflecting the practical impact of the “quantity-for-price” policy.

BTK inhibitors dominated the targeted treatment landscape for lymphoma, which is consistent with the high prevalence of B-cell lymphomas within non-Hodgkin’s lymphoma. Ibrutinib, as the first approved BTK inhibitor, served as the primary treatment option between 2018 and 2021. However, following its approval, zanubrutinib rapidly gained market share due to favorable clinical evidence, such as that reported in the ASPEN study (Dimopoulos et al., 2023), along with more competitive pricing. By 2022, zanubrutinib surpassed ibrutinib in prescription volume, reflecting the combined influence of efficacy, safety, and economic considerations in clinical decision-making. This shift highlights the growing preference for newer-generation BTK inhibitors in real-world practice.

Notably, orelabrutinib first appeared in the dataset in 2022, indicating that domestically developed innovative drugs in China are increasingly entering clinical use. As these agents continue to gain traction, the competitive landscape of the BTK inhibitor market may undergo further changes.

ALK inhibitors were used primarily for specific lymphoma subtypes, particularly anaplastic large cell lymphoma. Although their overall use showed an increasing trend initially, a gradual decline was observed in later years. Alectinib, as a second-generation ALK inhibitor, became the preferred option owing to its improved central nervous system penetration and enhanced therapeutic efficacy. After 2021, both the prescription volume and cost of alectinib exceeded those of crizotinib, whose use declined slightly, likely due to substitution by more effective alternatives.

This study also revealed that only 39.53% of patients were covered by medical insurance, with more than 60% bearing treatment costs out of pocket, underscoring the ongoing affordability challenges associated with targeted therapies. Although DDDc declined over time, the average annual treatment cost remained relatively high. From a policy perspective, medical insurance authorities should continue to improve evaluation frameworks that prioritize drugs with clear cost-effectiveness advantages while ensuring access to innovative treatments. The rapid rise of zanubrutinib further suggests that a balance of clinical efficacy, safety, and pricing is a critical determinant of market performance.

Several limitations should be acknowledged. First, prescription data were obtained from 77 hospitals in six major urban regions participating in the Hospital Prescription Analysis Cooperative Project. While these hospitals represent advanced healthcare settings, they may not fully capture prescribing patterns in rural or less developed areas. Second, the dataset reflects issued prescriptions but does not confirm patient adherence or treatment completion. Due to the limitations of data acquisition, we can only know that the patient’s diagnosis was lung cancer, but we cannot determine whether the lung cancer was the primary lesion or a secondary lesion.

Additionally, the sampling strategy relied on randomly selected days throughout each year, which may have resulted in the omission of certain prescriptions or drug combinations. The database did not support longitudinal tracking of individual patients, preventing analysis of treatment duration, sequencing, switching patterns, or long-term outcomes. Given the scale of the study, which encompassed prescription data from 77 hospitals and 1,232 outpatient visits across six major regions of China, it was not feasible to systematically assess overall survival, disease progression, or treatment adherence.

The prescription records reflect prescribing behavior only and do not provide information on actual medication compliance or clinical treatment outcomes. In addition, the database does not allow continuous tracking of individual patients over time, making it impossible to evaluate treatment duration, sequential therapy patterns, or treatment switching and conversion strategies. For some newly introduced drugs, the period between market launch and the end of the study was relatively short, and therefore long-term utilization trends and outcomes require further observation. Moreover, the data source, the Hospital Prescription Analysis Cooperative Project database, includes only 77 hospitals across six regions of China, which may limit the national representativeness of the findings, particularly for underdeveloped or rural areas.

Future research should aim to overcome these limitations by incorporating longitudinal, patient-level data and expanding geographic coverage to include a more diverse and representative population. Integrating prescription data with information on medication adherence, treatment response, and clinical outcomes would enable a more comprehensive evaluation of real-world effectiveness. In addition, large-scale prescription data analyses could provide valuable evidence to support clinical decision-making and policy formulation related to drug utilization and management. A nationwide assessment of small-molecule inhibitor use, incorporating adherence, therapeutic outcomes, and long-term follow-up data, will be essential to fully evaluate the clinical and pharmacoeconomic impact of these therapies in real-world settings.

## 4 Conclusion

This study systematically analyzed outpatient prescription data for lymphoma across six major regions of China from 2016 to 2022, revealing a sustained increase in the use of small-molecule targeted inhibitors alongside a gradual alleviation of patients’ economic burden. BTK inhibitors dominated the treatment landscape, with zanubrutinib showing rapid growth in clinical use due to its combined advantages in efficacy, safety, and cost, underscoring the growing importance of individualized treatment strategies and pharmacoeconomic considerations in lymphoma management. Despite the continued expansion of targeted therapy utilization, policy interventions such as medical insurance negotiations and centralized procurement have played a critical role in improving drug affordability without restricting access. Future studies should extend geographic coverage, integrate patient adherence and clinical outcome data, and conduct more comprehensive pharmacoeconomic evaluations to generate robust evidence supporting healthcare policy development and the optimization of clinical practice guidelines.

## Data Availability

The raw data supporting the conclusions of this article will be made available by the authors, without undue reservation.
